# Retrospective Evaluation of Urological Problems in Rare Childhood Syndromes

**DOI:** 10.7759/cureus.41577

**Published:** 2023-07-08

**Authors:** Sevim Yener, Ceyhan Şahin, Zekeriya Ilce

**Affiliations:** 1 Department of Pediatric Urology, University of Health Sciences, Umraniye Training and Research Hospital, Istanbul, TUR; 2 Department of Pediatric Surgery, University of Health Sciences, Umraniye Training and Research Hospital, Istanbul, TUR

**Keywords:** hydronephrosis, kidney, abnormality, urogenital, rare syndrome

## Abstract

Objective

Rare syndromes are defined as diseases that affect a small number of people compared to the general population. In the literature, rare syndromes have variable definitions according to countries. In this study, patients diagnosed with various rare syndromes who were referred to the pediatric urology clinic were examined in terms of associated urological anomalies.

Patients and methods

In this study, patients who were referred to our outpatient clinic between 2017 and 2022 with a diagnosis of a rare syndrome with or without urological or urogenital findings were retrospectively analyzed. The urinary system ultrasonography and scrotal ultrasonography of the patients were also recorded. Comorbidities, diseases, and surgeries they had undergone were determined through detailed medical history.

Results

A total of 32 patients were identified. Eleven (35%) of the patients were female and 21 (65%) were male. The average age of the patients was 6.5 years. The syndromes observed in the patients, in order of frequency, were microdeletion syndromes (n = 4), Roberts syndrome (n = 3), and Ehlers-Danlos syndrome (n = 2), and a variety of different syndromes were found in the remaining 22 patients. Despite having no symptoms, the second patient was found to have left hydronephrosis, and the third patient was found to have right renal ectopia on their urinary system ultrasound. Pathological findings were observed in 10 (31.2%) patients on their urinary system ultrasound.

Conclusion

Although only a small portion of these findings require surgery, the presence of urological anomalies should be investigated. Therefore, we recommend urological evaluation for all patients with rare syndromes, regardless of whether they have symptoms or not.

## Introduction

Rare syndromes are defined as diseases that affect a small number of people compared to the general population. In the literature, rare syndromes have variable definitions according to countries. Accordingly, in the United States, a disease that affects less than 200,000 people has been described as rare [1]. The European Union defines a disease or condition that affects less than one person in 2,000 in the general population as rare [2].

These syndromes can exhibit clinical features ranging from mild symptoms affecting only a part of the body to severe symptoms involving multiple organ systems. As there are some clinical findings in the diagnosis of rare syndromes, recent developments in medicine and genetic studies also contribute positively to the diagnosis process. Reports in the literature have indicated varying rates of urological anomalies associated with rare syndromes [3-5]. Some rare syndromes are characterized by intrauterine and/or postnatal growth retardation, mental retardation, skeletal malformation, and facial dysmorphism [6,7]. Hydronephrosis and renal ectopia, among the urinary system anomalies, and undescended testis and testicular agenesis can be seen if it is related to the genitourinary system. Although urological anomaly association has not been reported in some rare syndromes, it can be detected in case reports. It is known that the patient diagnosed with a rare syndrome should be examined in terms of the presence of another anomaly associated with it. From this point of view, we wanted to emphasize the importance of urological examination and evaluation in patients with rare syndromes.

In this study, patients diagnosed with various rare syndromes who were referred to the pediatric urology clinic were examined in terms of associated urological anomalies.

## Materials and methods

Patients who were referred from the pediatric clinic to the pediatric urology clinic of the Umraniye Education and Research Hospital between 2017 and 2022 and who were being followed up with a diagnosis of a rare syndrome were retrospectively examined. In our country, diagnosis, follow-up, and treatment of congenital anomalies and rare syndromes are covered by state insurance. The age, gender, reasons for referral to our clinic, and the rare syndromes they were being followed up for were determined. The syndromes were recorded according to the evaluation reports from the medical genetics departments of the patients. The urogenital examination findings of the patients were recorded. The sacral region was examined for hair growth, discoloration, sacral sinus, and sacral dimple. External genitalia examination, urethra-vaginal orifice opening in girls, testis examination in boys, and meatus location were evaluated. Urinary system ultrasonography was performed in all patients with or without urological symptoms. Scrotal ultrasonography was performed in male patients with examination findings such as undescended testis and/or size differences between testicles. Abdominal and/or pelvic ultrasonography was not performed for both patient groups in external genital examination (girls and boys) who did not reveal any finding such as bilateral non-papal testis, penoscrotal hypospadias, scrotalized labium, and/or single orifice in the introitus, suggesting ambiguous genitalia. Comorbidities, diseases, and surgeries they had undergone were determined through detailed medical history. As our study did not require any statistical calculation or comparison between groups, no specific statistical analysis was conducted. Our study received ethical committee approval from the Medical Ethical Committee of Umraniye Training and Research Hospital (approval reference number: 2022-E-54132726-000-18178).

## Results

A total of 32 patients were identified. Eleven (35%) of the patients were female and 21 (65%) were male. The average age of the patients was 6.6 (SD = 4.2) years. The syndromes observed in the patients, in order of frequency, were microdeletion syndromes (n = 4), Roberts syndrome (n = 3), and Ehlers-Danlos syndrome (n = 2), and a variety of different syndromes were found in the remaining 22 patients (Table 1).

**Table 1:** Demographic characteristics of the patients, syndrome diagnoses, urinary system anomaly, and USG findings U-USG: urinary system ultrasonography; S-USG: scrotal ultrasonography; USG: ultrasonography. Yes (+): There is a urinary system anomaly associated with the syndrome. No (-): No urinary system anomaly associated with the syndrome.

Patient number	Gender	Age (years)	Syndrome	Associated urinary system anomaly, (+) yes/(-) no	U-USG	Diagnosis and findings	S-USG
1	Male	6	Klippel-Feil syndrome	+	0		0
2	Male	1.5	Hennekam syndrome	+	0		Left undescended testicle
3	Male	1	Microdeletion syndrome (2q31,1 microdeletion)	0	0		0
4	Female	13	Seckel syndrome	0	Right renal agenesis		0
5	Male	5	Joubert syndrome	+	0		0
6	Male	4.5	Schinzel Giedion syndrome (2qdup-9pdel)	+	0		Bilateral undescended testicle
7	Female	8	Roberts syndrome	+	0		0
8	Male	4	Noonan syndrome	+	0		0
9	Male	2	Mucopolysaccharidosis type 2	0	0		0
10	Male	5	Mandibulofacial dysostosis Guion-Almeida type	0	0		Left undescended testicle
11	Male	2	Microdeletion syndrome [hg19](1-22)x2,(xy)x1	0	0		0
12	Male	10	West syndrome	0	0		Bilateral undescended testicle
13	Female	2	X-linked intellectual developmental disorder	0	Right hypoplastic kidney	Static scintigraphy renal function: right kidney function: 33.8%	0
14	Male	5	Microdeletion syndrome (13.1 microdeletion on chromosome 16)	0	0		0
15	Male	5	Roberts syndrome	+	0		0
16	Male	3	Robinow syndrome	+	Nephrocalcinosis of the left kidney	Hypercalciuria	0
17	Male	12	Cantu syndrome	0	Hydroureter in the left kidney	Nonobstructive non-reflux megaureter. Ureter diameter: 20 mm. Dynamic renal scintigraphy left kidney function: 48%. Voiding cystourethrography: no reflux	0
18	Female	8	Gitelman syndrome	+	Increase in bladder wall thickness	Dysfunctional voiding, daytime incontinence, and enuresis nocturna	0
19	Female	4	Dravet syndrome	0	Increased bilateral renal parenchymal echogenicity	Static scintigraphy renal function. Right kidney: 47%; left kidney: 53%	0
20	Female	8	Roberts syndrome	+	0		Hypoplastic uterus
21	Female	17	Wolfram syndrome	+	0		0
22	Male	5	Russell-Silver syndrome	+	0		Bilateral undescended testicle
23	Female	5	DiGeorge syndrome	+	0		0
24	Female	9	Williams syndrome	+	Hydronephrosis in the right kidney	Grade 1 hydronephrosis in the right kidney. Renal pelvis anteroposterior diameter 7.9 mm	0
25	Female	12	Smith-Magenis syndrome	0	Increase in bladder wall thickness	Dysfunctional voiding, daytime incontinence, and enuresis nocturna	0
26	Male	7	Tourette syndrome	0	0		0
27	Male	14	Kleefstra syndrome	+	0		Left undescended testicle
28	Male	1.5	Microdeletion syndrome (XP22-13 deletion)	0	Hydronephrosis in the left kidney	Left kidney grade 1 hydronephrosis. Pelvis anteroposterior diameter 5 mm	0
29	Male	3	Bardet-Biedl syndrome	+	0		Right testicle agenesis
30	Male	15	Cohen syndrome	0	0		0
31	Male	6	Ehlers-Danlos syndrome	0	0		0
32	Female	8	Ehlers-Danlos syndrome	0	Right renal ectopia	Renal ectopia located in posterosuperior right kidney	0

As a result of examinations and investigations, the following urinary system pathologies were identified: urinary incontinence (n = 13), undescended testicle (n = 8), interrupted urine flow (n = 3), buried penis (n = 2), neurogenic bladder (n = 2), vesicoureteral reflux (VUR) (n = 1), hypospadias (n = 1), and hydrocele (n = 1). Of the patients with incontinence, three had daytime urinary incontinence (overactive bladder), three had nocturnal enuresis, and seven had both daytime and nighttime urinary incontinence. An atypical posterior urethral valve was detected in a five-year-old male patient who had intermittent voiding. There was no abnormal finding in urinary system ultrasonography. Labial fusion was detected in one of the other two patients with intermittent voiding, and dysfunctional voiding was not detected in the other. A tethered cord was detected in the lumbar magnetic resonance imaging of two patients who were followed up for neurogenic bladder. The urodynamic finding was overactive and uninhibited detrusor in one patient, and normal capacity overactive detrusor contraction and postvoid residual urine in the other patient. A 13-year-old girl with a diagnosis of vesicourethral reflux had grade 2 reflux. She had frequent urinary tract infections. There was loss of function in the right kidney. As the first treatment, subureteric injection therapy was performed on the right side. Since urinary tract infection and reflux persisted in the follow-up, an open intravesical ureteroneocystostomy was performed.

Three patients were referred to us for screening purposes without any symptoms. The first patient was following due to Klippel-Feil syndrome, the second patient was under follow-up for microdeletion syndromes (XP22-13 deletion), and the third patient was following due to Ehlers-Danlos syndrome kyphoscoliotic type. Despite having no symptoms, the second patient was found to have left hydronephrosis and the third patient was found to have right renal ectopia on their urinary system ultrasound (US). Hydronephrosis grade was determined as grade 1. Conservative follow-up was done. Conservative follow-up was also performed on a patient with a diagnosis of posterior-superior renal ectopia. Pathological findings were observed in 10 (31.2%) patients on their urinary system ultrasound.

**Figure 1:**
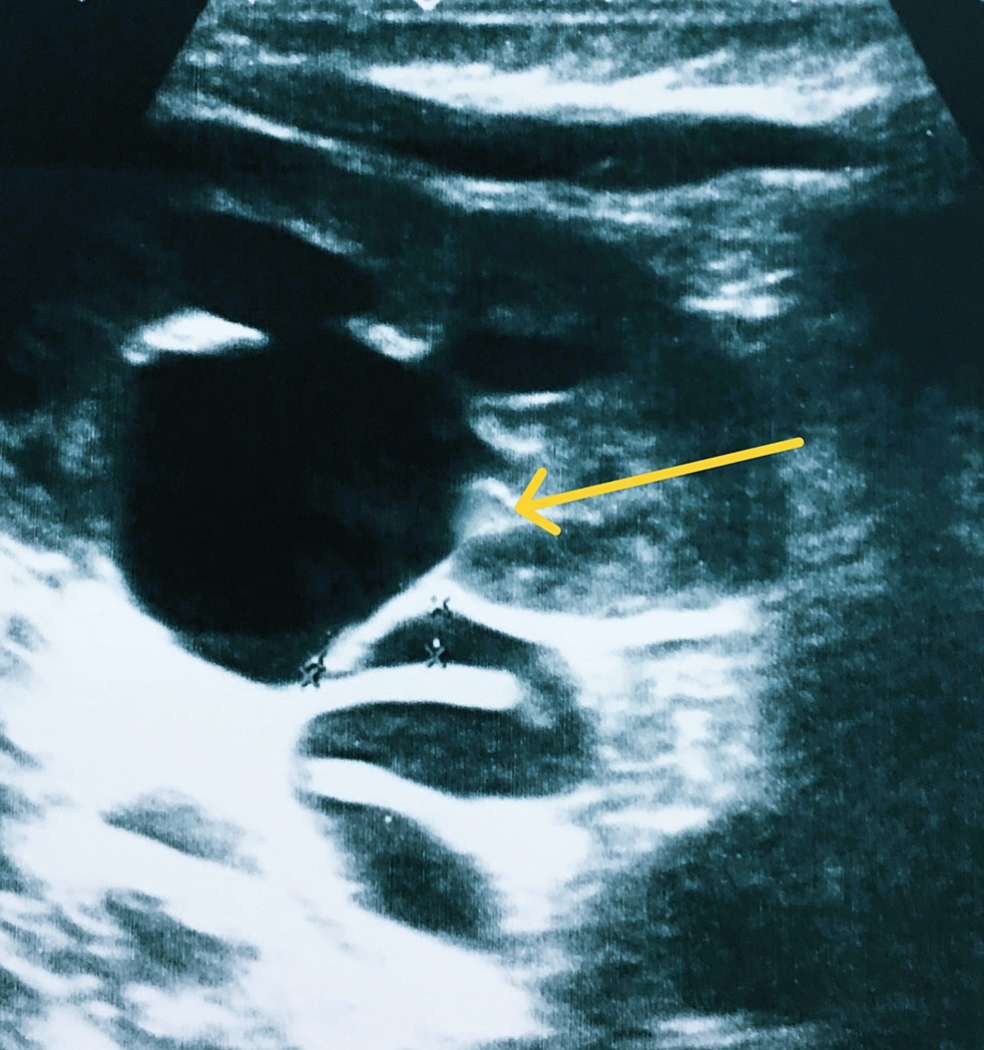
Hydroureteronephrosis. Dilated pelvicalyceal system marked with a yellow arrow

**Figure 2:**
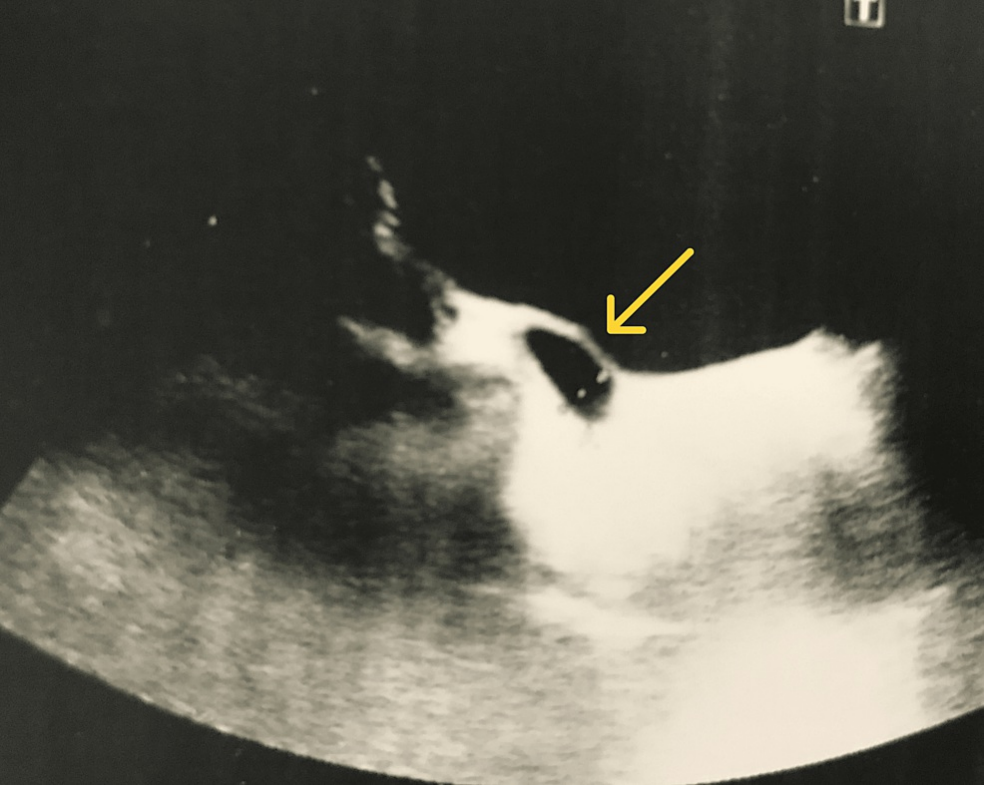
Hydroureteronephrosis. Dilated ureter marked with a yellow arrow

These included hydronephrosis (n = 3), renal agenesis (n = 1), renal hypoplasia (simple hypoplasia type) (n = 1), rotation anomaly (n = 1), hydroureteronephrosis (n = 1), nephrocalcinosis (n = 1), and increased parenchymal echogenicity (n = 1) (Figures 1, 2).

**Figure 3:**
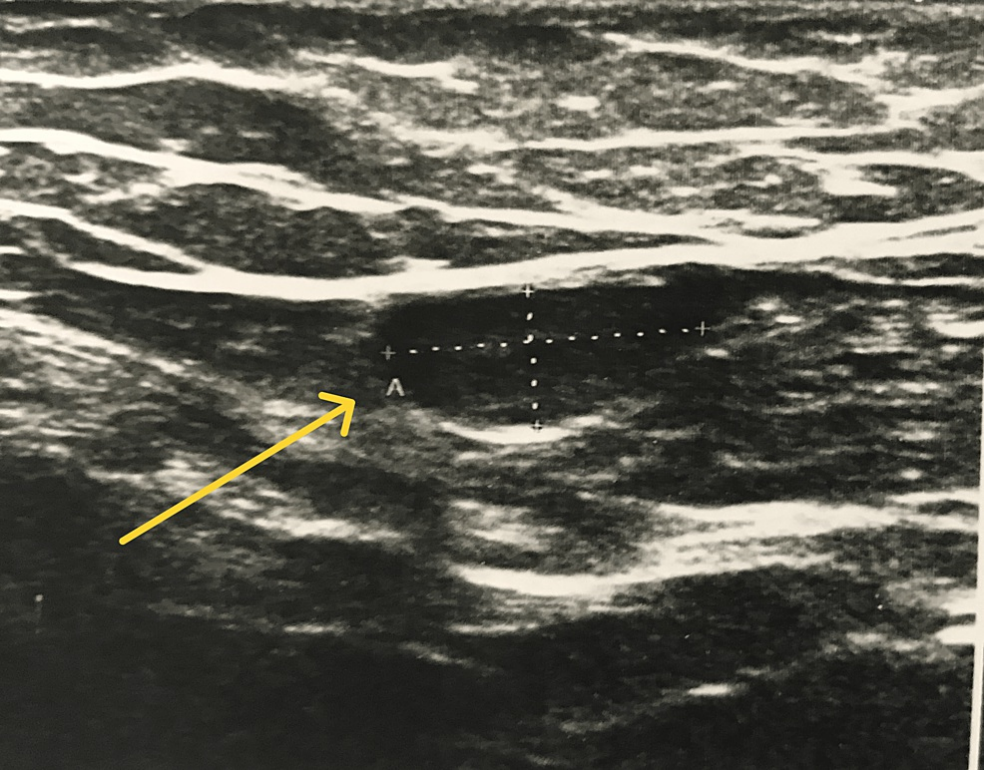
USG image of undescended testis. Testicular tissue is marked with a yellow arrow

Scrotal US revealed undescended testes in seven patients and hydrocele in one patient (Figure 3). The testicles of the patients were palpated in the inguinal canal.

When examined in terms of past surgeries, it was found that four patients underwent orchidopexy, three patients underwent high ligation, one patient underwent subureteric injection and subsequent ureteroneocystostomy due to vesicoureteral reflux diagnosis, one patient underwent nubbin testis excision, one patient underwent posterior urethral valve ablation, and one patient underwent buried penis surgery. The pathology result of nubbin testis excision was interpreted as aplasia since testicular tissue and hemosiderin accumulation and calcification findings were not observed.

## Discussion

Literature on rare syndromes is often presented in the form of case reports. Based on this, we examined the urological anomalies associated with the patients followed up for rare syndromes in our pediatric urology clinic. In our study, no urinary system anomaly was defined in the diagnostic parameters of the syndromes in which 16 (50%) patients were followed. However, in seven (43.75%) of these 16 patients, we found pathological findings in the urinary system US. Additionally, pathological findings were detected in the scrotal ultrasound of three of these 16 patients.

In other words, as a result of the USG examination of all patients, urinary system anomaly was detected in 10 patients. In three of these 10 patients, urological anomaly association was defined in the syndrome itself. However, the main point that attracted our attention was that urological pathologies were not defined as a component of the syndrome in which they were followed in the remaining seven patients.

These patients were evaluated individually, as listed below, in the order of their referral to our clinic.

Seckel syndrome

There is generally no recognized relationship between this syndrome and kidney disease [6]. In our patient with Seckel syndrome, right renal agenesis was detected, and there was vesicoureteral reflux to the same side. Initially, the injection was performed in the external center due to vesicoureteral reflux, and later ureteroneocystostomy was performed by open surgery. In a literature review, horseshoe kidney and kidney stones were reported in a 17-year-old male patient with a diagnosis of Seckel syndrome in a case report [7]. In another case report, a rare and sporadic renal malformation, oligomeganephronia, was reported in a patient with suspected Seckel syndrome with mosaicism in the 4th ring chromosome [8].

X-linked intellectual developmental disorder

In our case, the right hypoplastic kidney and rotation anomaly were detected. He was referred to our clinic with a diagnosis of anal atresia and neurogenic bladder. Genital anomalies such as hypospadias, undescended testis, and even ambiguous genitalia have been reported in X-linked intellectual developmental disorder. However, urinary anomaly association has not been reported [9].

Cantú syndrome

It is characterized by congenital hypertrichosis, distinctive coarse facial features, an enlarged heart with increased systolic function or pericardial effusion, and a large patent ductus arteriosus (PDA) requiring repair in many cases [10]. No reported urinary anomaly is observed. Our patient was referred to the clinic due to urinary and fecal incontinence, and left hydroureteronephrosis was observed during the urinary system screening.

Dravet syndrome

It is one of the most severe epilepsy syndromes in early childhood. It is characterized by hemiconvulsion or generalized clonic seizures triggered by fever in the first year of life, followed by myoclonic, focal, and generalized tonic-clonic seizures. Non-convulsive status epilepticus and epileptic encephalopathy are common [11]. A patient presenting with urinary incontinence was found to have an increase in the parenchymal echogenicity of bilateral kidneys on the urinary USG.

Smith-Magenis syndrome

It is a genetic disease characterized by facial dysmorphisms that become more prominent with age, developmental delays, cognitive impairment, characteristic behavioral patterns, and sleep disorders [12]. A patient with urinary incontinence was found to have an increase in the thickness of the bladder wall on USG.

XP22-13 deletion

XP22-13 deletion, a microdeletion syndrome, is a rare X-linked developmental disorder. Cataracts, dental anomalies, facial dysmorphisms, microphthalmia, microcornea, nystagmus, and strabismus can be seen [13]. Typically, patients have dental abnormalities, intellectual disability, and congenital heart disease [14,15]. Our patient with XP22-13 deletion had no symptoms and was referred to us for screening. Hydronephrosis was detected in the left kidney on the urinary system USG.

Ehlers-Danlos syndrome

It is a heterogeneous group of hereditary disorders characterized by abnormal collagen synthesis that affects the skin, connective tissues, joints, blood vessels, and other organs [16]. In our series, two patients were being followed up for Ehlers-Danlos syndrome. One of them presented with urinary incontinence, and no urinary system anomaly was detected on screening. The other patient had no symptoms. Our patient with familial Mediterranean fever (FMF), who was being followed up by the pediatric rheumatology clinic, was found to have right renal ectopia on the urinary system USG.

It is noteworthy that although the two patients in the last paragraph of our study had no symptoms, their urological findings were positive. The association of hydronephrosis, renal ectopia, and renal agenesis with these rare syndromes has not been described in the literature and may be coincidental or part of the syndrome. Therefore, it seems necessary to perform a detailed evaluation of the urinary system in patients being followed up for rare syndromes.

## Conclusions

We recommend urogenital screening for rare syndromes even if a previous urological anomaly has not been identified. Although only a small portion of these findings require surgery, the presence of urological anomalies should be investigated. Therefore, we recommend urological evaluation for all patients with rare syndromes, regardless of whether they have symptoms or not.

## References

[1] (2002). Rare Diseases Act of 2002. SEC. 3. NIH OFFICE OF RARE DISEASES AT NATIONAL INSTITUTES
OF HEALTH..

[2] (2020). European Commission. Rare diseases. https://research-and-innovation.ec.europa.eu/research-area/health/rare-diseases_en.

[3] Vega H, Gordillo M, Jabs EW (2023). ESCO2 Spectrum disorder. GeneReviews®.

[4] Frikha R (2020). Klippel-Feil syndrome: a review of the literature. Clin Dysmorphol.

[5] Altun E, Paydas S, Kaya B, Karayaylalı İ (2022). Wolfram syndrome: case report. (Article in Turkish). KSÜ Tıp Fak Der.

[6] Mann TP, Russell A (1959). Study of a microcephalic midget of extreme type. Proc R Soc Med.

[7] Jung M, Rai A, Wang L, Puttmann K, Kukreja K, Koh CJ (2018). Nephrolithiasis in a 17-year-old male with Seckel syndrome and horseshoe kidneys: case report and review of the literature. Urology.

[8] Anderson CE, Wallerstein R, Zamerowski ST, Witzleben C, Hoyer JR, Gibas L, Jackson LG (1997). Ring chromosome 4 mosaicism coincidence of oligomeganephronia and signs of Seckel syndrome. Am J Med Genet.

[9] Stevenson RE (2023). Alpha-thalassemia X-linked intellectual disability syndrome. GeneReviews®.

[10] Grange DK, Nichols CG, Singh GK (2023). Cantú syndrome. GeneReviews®.

[11] Connolly MB (2016). Dravet syndrome: diagnosis and long-term course. Can J Neurol Sci.

[12] Esener Z, Tekedereli İ (2018). A case of Smith-Magenis syndrome. (Article in Turkish). Ann Health Sci Res.

[13] Ding X, Patel M, Herzlich AA, Sieving PC, Chan CC (2009). Ophthalmic pathology of Nance-Horan syndrome: case report and review of the literature. Ophthalmic Genet.

[14] Gjørup H, Haubek D, Jacobsen P, Ostergaard JR (2017). Nance-Horan syndrome—the oral perspective on a rare disease. Am J Med Genet A.

[15] Coccia M, Brooks SP, Webb TR (2009). X-linked cataract and Nance-Horan syndrome are allelic disorders. Hum Mol Genet.

[16] Parapia LA, Jackson C (2008). Ehlers-Danlos syndrome--a historical review. Br J Haematol.

